# Construction and Optimization of an Ecological Network in Zhengzhou Metropolitan Area, China

**DOI:** 10.3390/ijerph19138066

**Published:** 2022-06-30

**Authors:** Jingeng Huo, Zhenqin Shi, Wenbo Zhu, Tianqi Li, Hua Xue, Xin Chen, Yanhui Yan, Ran Ma

**Affiliations:** 1 College of Geography and Environmental Science, Henan University, Kaifeng 475004, China; 15833699562@163.com (J.H.); zhuwb517@163.com (W.Z.); tianqi_li@henu.edu.cn (T.L.); xuehua15515683960@163.com (H.X.); chenxin6610090305@163.com (X.C.); yanyanhuisp@163.com (Y.Y.); m18790833632@126.com (R.M.); 2 Research Center of Regional Development and Planning, Henan University, Kaifeng 475004, China; 3 Key Laboratory of Geospatial Technology for the Middle and Lower Yellow River Region, Henan University, Ministry of Education, Kaifeng 475004, China

**Keywords:** ecological network, ecological source, ecological corridor, ecological node, Zhengzhou Metropolitan Area

## Abstract

Rapid urbanization aggravates issues related to protection and optimization of the ecological environment. Constructing an ecological network system, including ecological values in planning, and using landscape effects efficiently are important for adjusting regional ecological space and promoting local sustainable development. Land use data from eight time points between 1980 and 2020 in the Zhengzhou Metropolitan Area were used to identify the local ecological sources, corridors and nodes and to identify an ecological network with high structural integrity. The study used the FLUS, MSPA, MCR, and gravity models, hydrological analysis, and network structure evaluation by applying tools such as ArcGIS, Guidos Toolbox and Conefor. The results indicated that: (1) among the nine major ecological sources, those in the Yellow River Basin connected the large−scale sources in the east and west of the network, and the rest were located in the northeast, southeast and southwest of the research area, semi−enclosing the main urban area of Zhengzhou. (2) There were 163 least−cost paths and 58 ecological corridors, mainly distributed along the Yellow River Basin. (3) There were 70 ecological nodes, divided into 10 strategic, 27 natural ecological and 33 artificial environment nodes, distributed in key locations such as the core of each source and the intersection of corridors. (4) The ecological network included all the landscape elements in the research area and connected the main ecological substrates in a semi−enclosing network structure with one horizontal and two vertical corridors and four clusters.

## 1. Introduction

In recent years, Chinese cities have experienced a period of rapid development. The accelerating expansion of construction land is increasing the scope and degree of environmental impacts [1]. The expansion of urban space has further fragmented ecological sources and severely damaged the connectivity of regional landscapes [2]. In this context, rapidly expanding cities are faced with threats to ecological security, degradation of habitat quality and reduction of biodiversity. Recently, many studies have proposed the building of urban ecological networks to ease the conflict between urban expansion and habitat destruction [3]. The construction of ecological networks can not only promote the active circulation of ecological substances and energy in the city, but also plays an important role in planning urban ecological space and achieving regional sustainable development [4].

According to theories from landscape ecology, ecological networks use land resources to maximize ecological effects, which reflects the potential spatial pattern of ecological elements and functional structures [5]. An ecological network is composed of ecological sources, corridors, and nodes. It forms a composite system of biological flows by connecting ecological patches and is an effective method to improve the service value of ecosystems [6]. In the 1980s, ecological connectivity problems were widely explored in research world-wide, mainly focusing on the construction of ecological networks of nature reserves and national parks [7,8,9] and the ecological spatial planning of highly intensive land uses [10,11]. In the 1990s, China also began research into ecological networks, with the gradual formation of basic theories [12,13,14,15], methods and technologies [16,17,18], and practical applications [19,20,21,22]. After more than 30 years of development, systematic research on ecological networks has matured, with the establishment of an ecological space theory centered on networks and processes. Research methods include the percentage of importance of omitted patches (PIOP) [23], the landscape mechanism model [24], the integrated valuation of ecosystem services and tradeoffs tools (InVEST) [25], morphological spatial pattern analysis (MSPA) [26], spatial priority [27] and minimum cumulative resistance (MCR) [28]. In certain areas, cross-ecosystem research has been carried out on mountains [29,30], urban agglomerations [31,32], watersheds [33,34], provincial areas [35,36], urban areas [37,38,39] and county areas [40,41] on the basis of landscape ecological construction theory centered on human social activities. Ecological planning theories focusing on maximizing multiple ecological values were adopted for research into land and marine planning [42,43], forest landscapes [44,45], ecosystem services change in an oasis [46], evaluation of ecologically sensitive areas [47], river bank buffering areas [48] and urban ecological landscapes [49,50,51].

With the fast pace of urbanization, the substantial impact of human activities and resource use on ecological space has become a research focus in terms of regional landscape ecology [52]. The ecological value system is dynamic. To maintain ecological values, it is important to build a regional ecological network structure, systematically identify ecological sources, corridors and nodes, and deal with land use changes, with the goal of control and guidance [53,54,55]. In previous studies [37,38,39,40], ecological networks were constructed on the basis of past ecological sources, but less attention was paid to the impact of resistance or facilitation factors during ecological development, which resulted in a lack of guidance and planning in the research results. At the same time, corridors and nodes in ecological networks tend to be simple overlays of elements in most studies [46,55]; thus, there are problems such as excessive subjectivity. In this paper, future ecological sources were introduced into the research through methods such as simulation. Ecological elements were systematically selected to improve the science and sustainability of network construction by considering the influencing factors of future spatiotemporal driving factors and applying a hierarchical evaluation index system.

The ecological space of the Zhengzhou Metropolitan Area is a patchwork of many complex ecological elements, and its landscape connectivity is interrupted by urban construction to a large extent. At present, there are plans for ecological protection and high-quality development in the Yellow River Basin, which has a positive effect on environmental improvement in the research area; however, the plans are missing an explanation of the circulation and structure of ecological energy within the metropolitan area.

This research focuses on the Zhengzhou Metropolitan Area. The land use data for eight time points between 1980 and 2020 were selected for the planning and construction of an ecological network. First, the FLUS−Markov coupling model was applied to predict and simulate the land cover in 2030 and 2035, and the simulation results were used as basic land use data for later analysis. The MSPA method was adopted to extract ecological landscape elements and screen the optimal distance threshold to identify the final ecological source sites in combination with landscape connectivity. Coupled with MCR and the gravitational model, the final ecological corridor is extracted by index evaluation with the least cost path as the main analysis content. The ecological corridors are combined with the hydrological analysis results of the cost resistance surface to jointly determine the critical ecological nodes. The ecological network of the study area is constructed based on the results of the final source, corridors and nodes, and the structural evaluation index of the network is used to establish a guidance planning system. This study respects the law of natural development and aims at sustainable regional development, providing a scientific basis for ecological spatial planning and future ecological network optimization directions in the Zhengzhou Metropolitan Area.

## 2. Study Area and Material

### 2.1. Study Area

**Figure 1:**
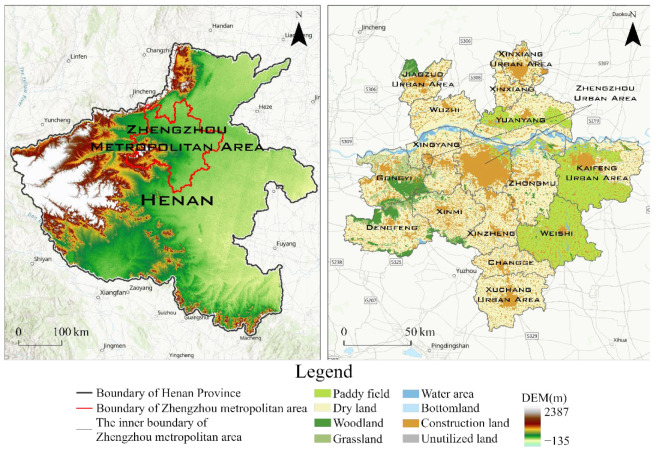
Geographical location of Zhengzhou Metropolitan Area.

The administrative scope of the Zhengzhou Metropolitan Area includes the cities of Zhengzhou, Gongyi and Changge, the central urban areas of Kaifeng, Xinxiang, Jiaozuo and Xuchang, and the urban−rural integration demonstration areas of Wuzhi, Yuanyang, Xinxiang, Weishi and Pingyuan (Figure 1). The study area covers 1.59 million km^2^, accounting for 9.6% of the land area of Henan Province, approximately 20% of the province’s population and more than 30% of the total economic output. In 2020, its GDP was USD 232.30408 billion, with a total population of 16.7253 million and an average urbanization rate of 55.18%. The area is in the middle and lower reaches of the Yellow River and the central and northern parts of Henan, at longitude 112°42′–114°50′ E and latitude 33°51′–35°26′ N. Owing to its location in a warm temperate zone, it has an average annual precipitation of 700−900 mm. The region is characterized by high terrain in the west and low terrain in the east, and it is adjacent to Taihang Mountain in the north and Song Mountain in the west. Lying in the Land Bridge Passage and the intersection of the Beijing−Harbin and Jing−Guang Passages in China’s “east–west” and “north–south” throughout urbanization strategy, it is the most dynamic area of development in the New Eurasian Continental Bridge Economic Corridor. However, the vigorous promotion of urban development and construction has resulted in substantial loss of natural land cover and poses a threat to the local ecosystem. In this context, the construction and optimization of an ecological network in the Zhengzhou Metropolitan Area will support regional sustainable development.

### 2.2. Data and Preprocessing

The data involved in this research included land use/cover data, a digital elevational model (DEM), slope, road network, rivers, points of interest (POI), local planning content and species-related information for the Zhengzhou Metropolitan Area. (1) Land use/cover data: 30 m × 30 m resolution land use data in 1980, 1990, 1995, 2000, 2005, 2010, 2015 and 2020. The original data were divided into eight land types: paddy fields, dry land, woodland, grassland, water area, bottomland, construction land and unutilized land. The data came from Resource and Environment Science and Data Center (http://www.resdc.cn/, accessed on 3 April 2021). (2) DEM and slope data: the DEM data came from the Geospatial Data Cloud, and the slope was calculated with the DEM (http://www.gscloud.cn/, accessed on 3 April 2021). (3) Data for the road network, rivers and POI: four types of road information, including railways, expressways, and national and provincial roads in 2019 were extracted, and rivers above grade 3 were selected as the main research objects. The POI included forest parks, natural scenic spots and mountains, and the data came from OpenStreetMap (http://www.openstreetmap.org/, accessed on 5 April 2021). (4) Planning content and species-related information: the ecological protection red line and nature reserves were used as the main reference content to match the actual ecological environment effects and meet the requirement of species diffusion. The relevant content came from The Spatial Planning of Zhengzhou Metropolitan Area (2018–2035) (https://fgw.henan.gov.cn/, accessed on 15 April 2021). The geographic elements involved in the research were uniformly processed with a spatial resolution of 30 m × 30 m, using the WGS 1984 World Mercator projection coordinate system.

## 3. Methodology

**Figure 2:**
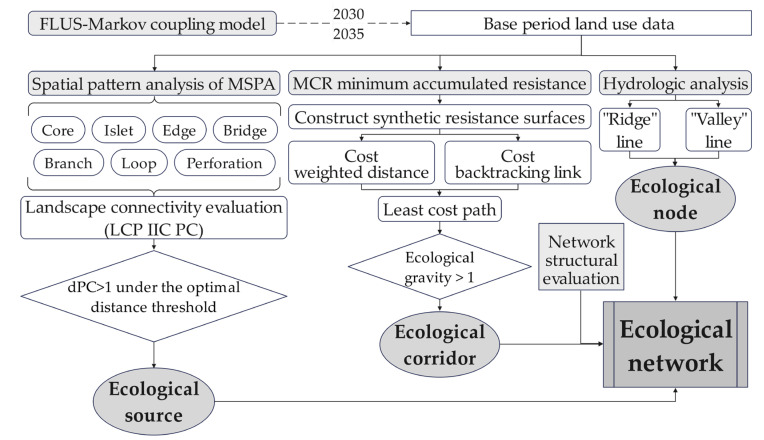
A flow chart depicting the analytical process of the research methodology.

In the research, first, the land cover in 2030 and 2035 predicted by the FLUS–Markov coupled model and the existing land cover from 1980 to 2020 were used as the basic land use data. Then, seven landscape elements, including core area, isolated island, edge area, bridge area, branch line, circle and pore, were identified by MSPA. The landscape coincidence probability (LCP), integral index of connectivity (IIC), probability of connectivity (PC) and optimal distance threshold were used to determine the ecological source area. Then, a comprehensive resistance evaluation system was constructed, a comprehensive resistance surface was built by MCR, and the minimum cost path was obtained by cost distance calculation. A gravity of >1 was selected as the optimal cumulative cost path, namely the ecological corridor between the source areas. Hydrological analysis was used to calculate a comprehensive resistance surface, and the intersections of the ridge lines and valley lines were screened. Roads, rivers and other factors were introduced to identify ecological nodes. To summarize, the ecological network system of Zhengzhou Metropolitan Area was constructed by spatially overlaying ecological sources, ecological corridors and ecological nodes. The ecological network was tested and evaluated using network structural analysis. The method and workflow are shown in Figure 2.

### 3.1. FLUS−Markov Coupling Model

The FLUS model is used to couple human activities and the natural environment on the basis of land use conditions and to simulate and predict future land demands in the face of spatio-temporal dynamics [31]. The Markov chain prediction method is applied to predict the number of pixels of land changes in a period according to the existing land changes [37]. The coupled model can calculate the probability of land suitability using a back propagation−artificial neural network and gradually make land changes to approach a target result within a preset land demand threshold by conducting multiple iterations through the spatial configuration of cellular automaton.

In the current study, according to the long-term spatial planning of the Zhengzhou Metropolitan Area, the land use data from 2010 to 2020 were used to predict the land use in 2030 and 2035 by introducing various driving factors such as elevation, slope, roads, rivers, population density and residential areas. The simulation results were used as the land use data in the base period to construct the ecological network, to ensure precise and accurate future simulations. The specific parameters are shown in Table 1.

### 3.2. Morphological Spatial Pattern Analysis

**Figure 3:**
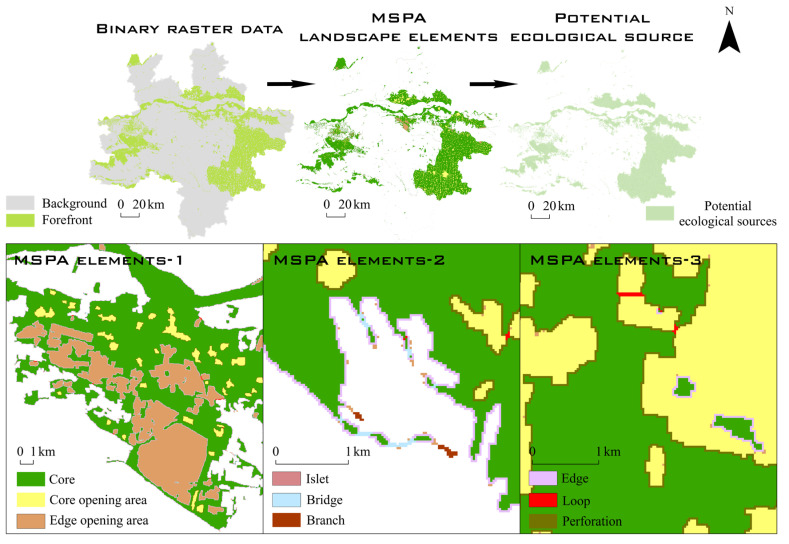
MSPA process and ecological meaning of MSPA types.

MSPA was used to measure, select, and segment the spatial forms of raster images on the basis of morphological principles such as erosion, dilation, and opening and closing operation, to precisely divide landscape structures and types [26]. In this research, paddy fields, woodland, grassland, water area, and bottomland were selected as foreground files, and the rest were classified as background files. All of them were converted into binary raster data in TIFF format. The Guidos Toolbox software was applied with the foreground connection set to 4, the edge width to 1, the conversion rate to 1, and the opening to 1. An eight-neighborhood analysis method was adopted for the MSPA to obtain seven types of landscape elements (Table 2) in the research area, and the core area blocks were extracted as potential ecological sources (Figure 3).

#### 3.2.1. Landscape Connectivity Assessment

Landscape connectivity is a measure of the spatial continuity between ecological structural units [27,35]. In this research, the LCP index (Equation (1)) was selected to reflect the overall coherence between ecological components, the IIC (Equation (2)) was used to express the overall stability of the habitat, and the PC index (Equation (3)) was used to reflect the overall connectivity between blocks. To distinguish the importance of different patches, the dΦ index (Equation (4)) was selected to evaluate the contribution of patches to the overall landscape connectivity.

(1) $$\mathrm{LCP}=\overset{\mathrm{NC}}{\underset{i=1}{\sum }}{\left(\frac{\Delta_{i}}{S_{L}}\right)}^{2}\;$$

(2) $$\mathrm{IIC}=\frac{\sum_{i=1}^{n}\sum_{j=1}^{n}\frac{a_{i}{.a}_{j}}{{1+n\xi }_{\mathrm{ij}}}}{S_{L}^{2}}$$

(3) $$\mathrm{PC}=\frac{\sum_{i=1}^{n}\sum_{j=1}^{n}a_{i}{.a}_{j}{.\mu }_{\mathrm{ij}}^{*}}{S_{L}^{2}}$$

(4) $$d\Phi =\frac{{\Phi \;–\;\Phi }_{\mathrm{remove},\;x}}{\Phi }$$

where NC (number of components) refers to the whole part composed of interconnected blocks; $\Delta_{i}$ is the sum of the area of the landscape components; $S_{L}$ is the total area of blocks; n is the total number of blocks; $a_{i}$ and $a_{j}$ are the areas of blocks i and j, respectively; ${n\xi }_{\mathrm{ij}}$ is the number of connections on the shortest path between blocks i and j; $\mu_{\mathrm{ij}}^{*}$ is the maximum connection probability between i and j; Φ is the result of a connectivity index; $\Phi_{\mathrm{remove},\;x}$ is the result of the connectivity index after removal of the x-th block.

#### 3.2.2. Distance Threshold Calculation

On the basis of the habitat range of mammals and the flight paths of birds in the research area, the potential ecological source areas in 1990, 2005 and 2020 were selected, with five distance thresholds, including 100 m, 500 m, 1000 m, 1500 m and 2000 m. The connectivity probability was set to 0.5, and the connectivity index was calculated by Conefor 2.6 software (http://conefor.org/ accessed on 1 October 2021). The connectivity index was used to measure the optimal distance threshold (Table 3). The optimal distance threshold was 1000 m, with a connectivity probability of 0.5. On this basis, the landscape connectivity indexes of potential ecological sources from 1980 to 2035 were re-calculated to select the ecological blocks with a dPC > 1 as ecological sources.

### 3.3. Minimum Cumulative Resistance Model

The MCR model (Equation (5)) measures the cumulative cost between ecological sources and target sources to express the spatial connectivity and accessibility between sources and obtain the optimal diffusion path for the migration of biological species [28].

(5) $${\mathrm{MCR}=F}_{\min}\sum_{i=1}^{m}\sum_{j=1}^{n}{(\mathrm{Dis}}_{\mathrm{ij}}{\cdot \theta }_{i})$$

where MCR is the cumulative value of the minimum resistance between ecological source j and any ecological source i; ${\mathrm{Dis}}_{\mathrm{ij}}$ is the spatial distance between i and j; $\theta_{i}$ is the resistance coefficient of the ecological source i to the ecological flow; $F_{\min}$ is the positive correlation between minimum cumulative resistance and the ecosystem.

#### 3.3.1. Least-Cost Path

Materials and energy need to overcome different expansion resistances to flow between ecological environments. Constructing an ecological resistance surface can reflect the degree of obstruction to ecological connectivity [52].

**Figure 4:**
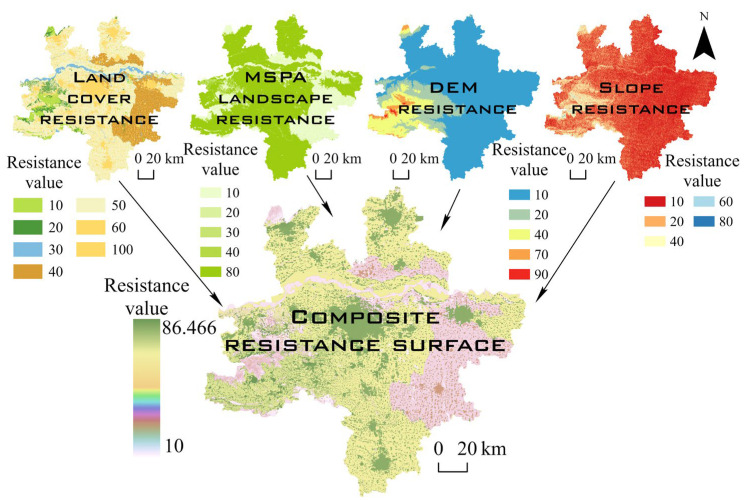
Synthesis of resistance surface formation.

The basic resistance coefficient was set between 1 and 100 in this paper (Table 4) on the basis of other studies [34,43]. The terrain data (DEM and slope) were divided into five levels of resistance using the natural breaks method (Figure 4). Land cover types and MSPA analysis results were graded in accordance with the strength of the ecological effect and the importance of landscape factors. The weight of the resistance layer was assigned using the analytic hierarchy process (AHP). The grid calculator was used to calculate the overlaid value of the 26 evaluation factors to create the comprehensive resistance surface for the 10th period from 1980 to 2035.

**Figure 5:**
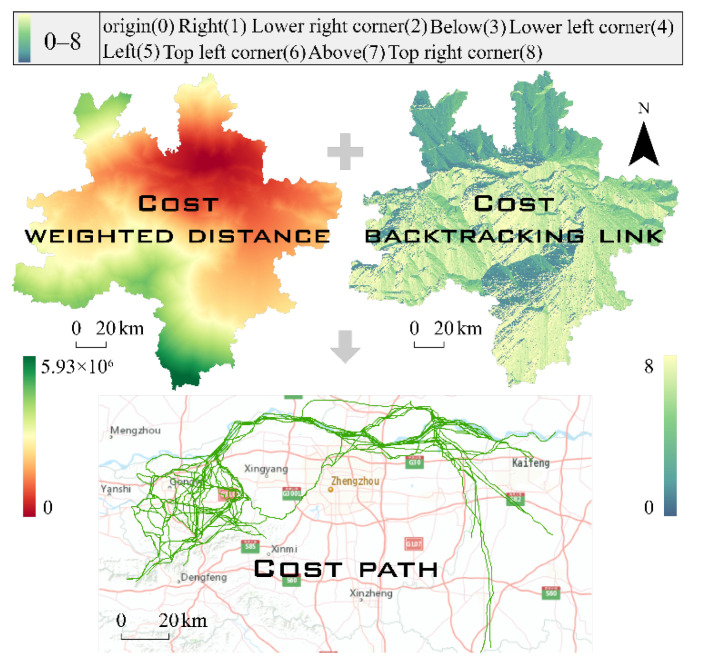
Cost path formation.

The least-cost path between ecological sources can fully reflect the permeability of corridors [39]. The ArcGIS cost distance module was used to generate a weighted cost distance and cost backtracking connection file by combining the ecological sources and comprehensive resistance surface. The cost path tool was applied to calculate the least-cost path from the source to the target, thereby identifying potential ecological corridors after the removal of redundant paths (Figure 5).

#### 3.3.2. Gravity Model

The gravity model can quantitatively evaluate the interaction strength between the source and target source with the cumulative resistance value. It is usually applied to judge the relative importance of ecological corridors [51]. The larger the ecological gravity value (Equation (6)) in the model, the smaller the resistance value between the ecological sources, which means that the importance of the ecological corridor connecting them is stronger.

(6) $${F=G}_{\mathrm{ij}}=\frac{\Omega_{i\cdot }\Omega_{j}}{{Ɩ}_{\mathrm{ij}}^{2}}=\frac{\frac{{\mathrm{lnS}}_{i}}{{Ɠ}_{i}}\cdot \frac{{\mathrm{lnS}}_{j}}{{Ɠ}_{j}}}{{\left(\frac{L_{\mathrm{ij}}}{L_{\max}}\right)}^{2}}=\frac{L_{\max}^{2}{\mathrm{lna}}_{i}{\mathrm{lna}}_{j}}{L_{\mathrm{ij}}^{2}}\;$$

where F is the ecological gravity; $G_{\mathrm{ij}}$ is the ecological gravity between ecological sources i and j; $\Omega_{i}$ and $\Omega_{j}$ are the block weights of i and j; ${Ɩ}_{\mathrm{ij}}$ is the normalized value of potential corridor resistance between i and j; $S_{i}$ and $S_{j}$ are the areas of i and j; ${Ɠ}_{i}$ and ${Ɠ}_{j}$ are the block resistance values of i and j; $L_{\mathrm{ij}}$ is the cumulative resistance of the corridor connecting i and j; $L_{\max}$ is the maximum resistance value in each corridor.

### 3.4. Hydrological Analysis

Ecological nodes are key locations in the process of biological diffusion or movement and areas where the ecological flow is vulnerable to obstructions [31,49]. In the current study, the ArcGIS hydrological analysis module was used to calculate the ridge lines and valley lines from the MCR surface and combine these with the ecological corridors to identify the ecological nodes.

The key ecological nodes were divided into strategic nodes, natural ecological nodes and artificial environment nodes in relation to the landscape matrix in landscape ecology [34] and considering human social activities and natural environment factors. The intersections between ridge lines with the maximum cost path and ecological corridors were designated as strategic nodes, which were the key areas for ecological improvement. The intersections between ecological corridors that persisted between 1980 and 2035 were designated as natural ecological nodes. These act as stepping stones for biological diffusion, material exchange and landscape connection in the corridor network. The intersections between the valley lines and the roads, rivers and paddy fields in the research area were designated as artificial environment nodes. These are the main areas where biological flows are obstructed artificially or naturally.

### 3.5. Network Structural Evaluation

In the current research, network structure analysis was applied to comprehensively evaluate the modeled ecological network in the Zhengzhou Metropolitan Area and optimize the ecological network pattern with the greatest ecological benefits [43].

The network closure degree (α index), node connectivity rate (β index), network connectivity (γ index) and cost ratio index were selected to conduct structural analysis on the ecological network. When the α index (Equation (7)) ranges from 0 to 1, the larger the value, the better the network liquidity. The larger the β index (Equation (8)), the higher the complexity of the network connection. The γ index (Equation (9)) represents the corridor connection ratio between ecological nodes; thus, the larger the value, the higher the node connectivity. The cost ratio index in the research represents the number and scale of ecological corridors across counties.

(7) $$\alpha =\frac{L-V+1}{2v-5}$$

(8) $$\beta =\frac{L}{V}$$

(9) $$\gamma =\frac{L}{3(v-2)}$$

(10) $$\mathrm{Cos}\mathrm{tratio}=1-\frac{L}{C}$$

where L is the number of ecological corridors; V is the number of ecological nodes; C is the length of a corridor.

## 4. Results

### 4.1. Identification of Ecological Sources

**Figure 6:**
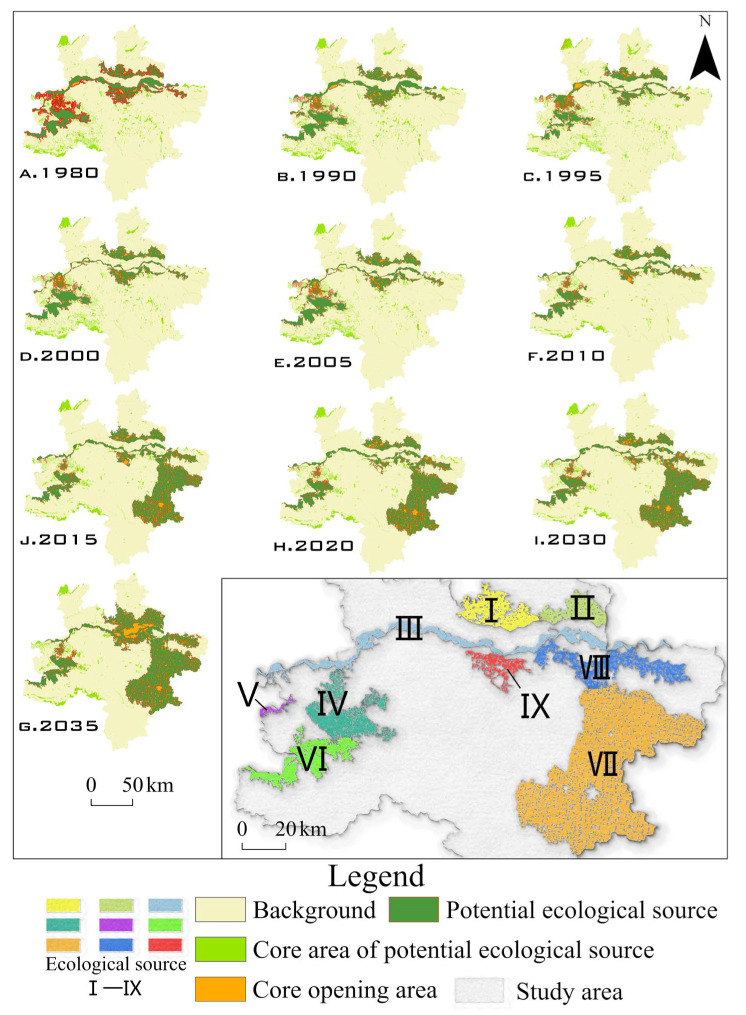
Ecological source identification using MSPA.

According to the MSPA (Figure 6), the sources with the strongest connectivity in the landscape were mostly concentrated in the buffer zone along the Yellow River Basin. Gongyi and Dengfeng in the north and southwestern regions, respectively, were important ecological sources but were characterized by substantial fragmentation. Owing to their proximity to the urban area of Jiaozuo, the potential sources identified in the northwest of the research area have substantial transmission resistance, and their ecological effect is greatly reduced because of the long distance to the core area of the Zhengzhou Metropolitan Area; thus, they were not selected as sources in the final model. Ecological sources began to increase substantially, and paddy fields in Kaifeng and Weishi County were included as important ecological sources.

On the basis of landscape analysis in 2030 and 2035, the predicted ecological sources gradually expanded from their original area, occupying 20.46% and 26.48% of the research area. This substantially reduced landscape heterogeneity and enriched the number of landscape types.

Table 5 indicates that most ecological sources were paddy fields, woodland, grassland and water areas. Paddy fields increased by 2991.64 km^2^, which was the maximum transfer area. The larger area of paddy fields provided a land use type in the Zhengzhou Metropolitan Area that could adjust the ecological functions and optimize the ecological structure. Woodland, grassland, and water area, with increases of 344.02 km^2^, 305.7 km^2^ and 406.16 km^2^, respectively, were widely distributed. They were the main objects identified as ecological sources and played an important role in the diversity of elements in the ecological network. The change in the area of bottomland was 126.31 km^2^, making it the smallest land type year-on-year.

### 4.2. Extraction of Ecological Corridors

According to the constructed ecological resistance surface, the north central, southwestern and eastern parts of the research area had low resistance, and these were the main areas connecting ecological corridors. Zhengzhou, Zhongmu, Xinmi, Xinzheng, Changge and Xuchang were located on flat terrain, and they had a higher proportion of construction land and dry land than those of other areas. This caused more ecological resistance in the central and southern parts of the research area.

The least-cost paths from 1980 to 2035 were extracted on the basis of the MCR analysis and the influence of rivers and roads in the research area. A total of 163 potential ecological corridors were identified after the exclusion of paths less than 10 km long and duplicated paths. The gravity model was applied to judge the relative importance of the potential corridors in each period (Table 6).

A total of 58 paths were finally identified as ecological corridors and divided into three levels after the exclusion of the corridors whose ecological gravity was <1 (Table 7). A total of 10 corridors with a gravity above 100 were rated as level 1. A total of 19 corridors with a gravity between 10 and 100 were rated as level 2. A total of 29 corridors with a gravity between 1 and 10 were rated as level 3.

**Figure 7:**
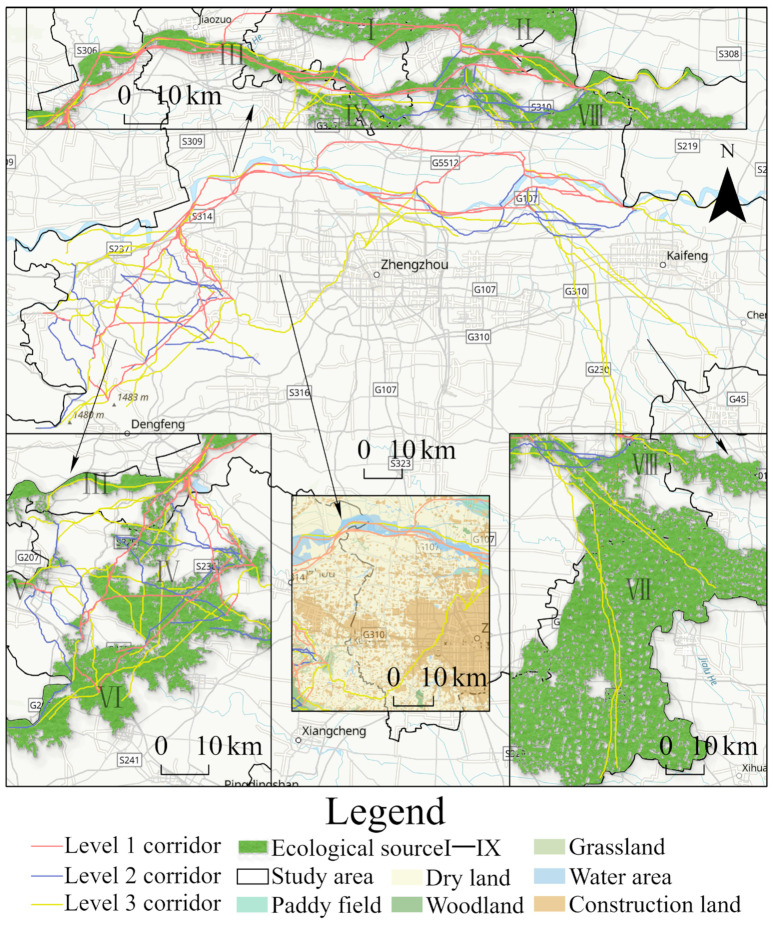
Extraction and classification of ecological corridors.

(1) The level 1 corridors passed through Yuanyang, Wuzhi, Xingyang, Gongyi and Dengfeng (Figure 7), mainly lying along the Yellow River Basin and running through the eastern and western parts of the research area. Five of the level 1 corridors were over 300 km, mainly connecting woodland, grassland, water area and the opening areas of paddy fields as well as all important ecological sources.
(2) Level 2 corridors were mainly distributed close to level 1 corridors, extending outwards on this basis. They connected with other corridors to form the spatial prototype of the ecological network. Corridor 11 was the longest level 2 corridor, at 455.11 km. It was mainly distributed along the periphery of the Yellow River.
(3) Of the level 3 corridors, 21 were less than 100 km, and they supplemented the netlike elements of the ecological network. They mainly formed a mountain corridor network in the Gongyi District, a circular corridor structure in northwest Zhengzhou, and a “pole-axis” corridor pattern in the Kaifeng area. Corridors 40–49 ran through the whole of Gongyi, showing a spatial distribution of three horizontal and three vertical corridors. Corridors 30−37 ran through the Huiji District, Zhongyuan District, Shangjie District and Xingyang City in Zhengzhou and were the only group that passed through a large area of construction land. To the north of Zhongmu, Corridors 41–50 radiated towards Kaifeng City and Weishi County, mainly connecting the paddy fields in the research area.

### 4.3. Determination of Ecological Nodes

**Figure 8:**
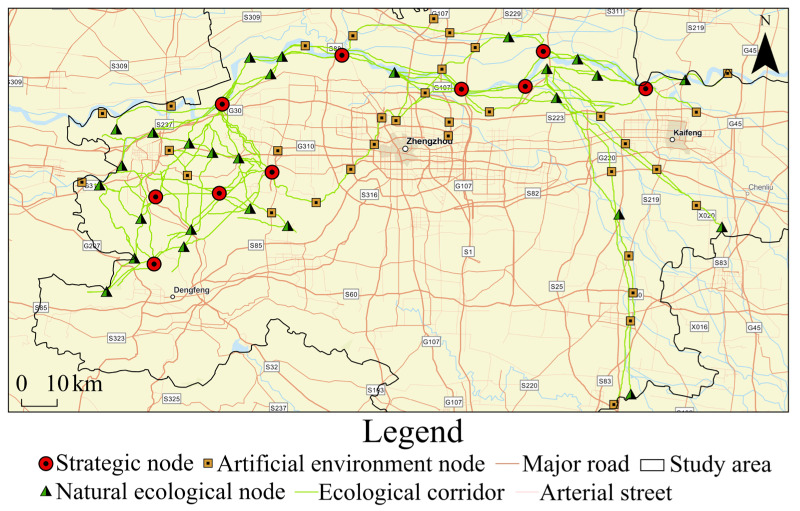
Determination of ecological nodes.

Ecological sources and ecological corridors were initially identified to form the basic framework of the regional ecological network. The ecological nodes serve as stepping stones to ensure the smooth operation of ecological flows. A total of 70 ecological nodes were identified in this research, including 10 strategic nodes, 27 natural ecological nodes and 33 artificial environment nodes (Figure 8).

The strategic nodes were distributed from the northwest of Kaifeng to the northwest of Xingyang and connected in Gongyi, Xinmi and Dengfeng to form one horizontal and three vertical level 1 ecological corridors. They were widely distributed in the III−V, VII and IX sources with high ecological value, but they were scarce in the I, II and VI sources. Natural ecological nodes were concentrated on the periphery of medium-sized ecological sources, and they were also the intersections of densely packed level 2 ecological corridors. Only three nodes were dispersed in VI sources, which were mostly paddy fields. The artificial environment nodes were mostly road and park nodes. The paddy fields were widely distributed in the urban area and southeast of Zhengzhou and were greatly affected by human factors. In addition, they tended to be located less near woodland and water areas and more in the IV sources.

### 4.4. Construction of Ecological Network

According to the evaluation of the network structure (Table 8), the α index of the ecological network constructed in this paper ranged from 0.07 to 0.45, indicating that ecological materials had lower circulation in the strategic nodes but that a combination of ecological nodes and level 2 and level 3 corridors had a positive effect on ecological material circulation. The β index ranged from 1 to 1.78, indicating the high complexity of the network and the large flexible space for ecological restoration. The γ index ranged from 0.42 to 0.64, indicating the tight connection in some locations and the large gap in ecological elements in the southern part of the network. The cost ratios were all above 0.98, indicating the large number of corridors connecting different administrative areas and the strong cross-regional circulation of ecological elements.

**Figure 9:**
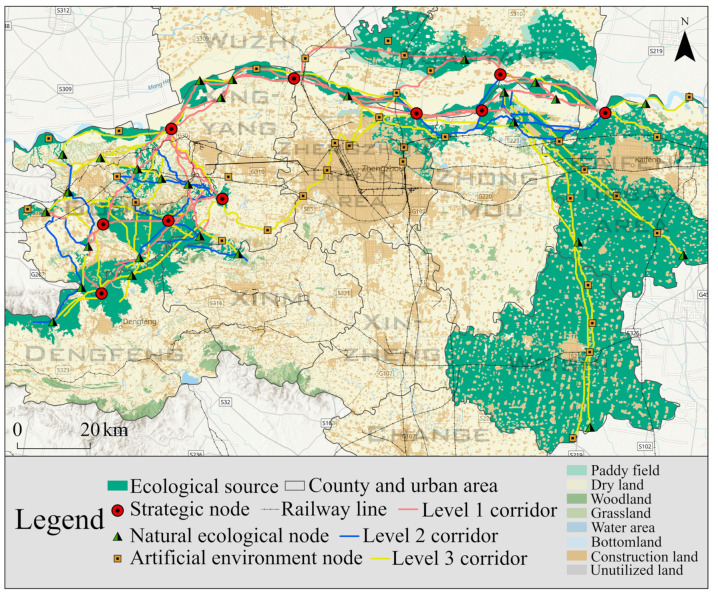
Construction of the ecological network.

As a whole, the ecological network semi-enclosed Zhengzhou, presenting a C-shaped structure (Figure 9). The east and west of the study region were mostly mountains, forest parks and farmland blocks, which formed a secondary network. The Yellow River Basin was the main channel connecting various ecological areas. The western mountain areas had a higher ecological suitability, but they were characterized by more substantial fragmentation. The secondary ecological network in this area connected the local forest parks and ecological sources. Some level 3 corridors and artificial environment nodes ran through the construction land in the central and southern parts of the research area. As ecological arteries in urban construction, they connected green spaces such as the Zhengzhou Pet Park, Farmer Park, Tianjianshan Park and Yunmengshan Park.

## 5. Conclusions and Optimization Strategies

### 5.1. Conclusions

In this study, the ecological network system of the Zhengzhou Metropolitan Area was constructed on the basis of spatial planning and landscape ecology, including nine ecological sources, 58 graded ecological corridors and 70 diverse ecological nodes. The results provide new ideas for improving the stability of the ecological pattern, optimizing the structure and function of the ecological environment, and planning for future ecological space. The research indicates the following:

(1) Among the nine major ecological sources, the Yellow River Basin was the only one with a long narrow structure that connected the large-scale ecological sources in the east and west of the network. The remaining sources were concentrated large-area blocks and were distributed in the northeast, southeast and southwest of the research area. These blocks played a role in storing energy, providing habitats for creatures and improving connectivity for ecological flows. The semi-enclosing distribution structure of the nine major sources is important for controlling the future development of the ecological pattern.
(2) Among the 58 ecological corridors, the level 1 corridors overlapped with the Yellow River Basin. With the river’s ecological advantages, these corridors had the largest influence range in the network system. A secondary network with three horizontal and three vertical corridors was formed in the southwestern part of the research area, which had the most complex structure in the network system. The southeastern part was mostly level 3 corridors, which ran toward paddy fields in a pole-axis pattern.
(3) The 10 strategic nodes were scattered across key areas of the ecological network. Most of them were intersections between the source openings and corridors. Within the core areas of the sources, there were 27 natural ecological nodes, which were key nodes in the natural landscape structure. The 33 artificial environment nodes were mainly situated at the intersection between corridors and existing parks, roads and rivers. They were typical representatives of the harmonious development of the artificial environment and natural ecology.
(4) The ecological network contained landscape elements such as paddy fields, woodland, grassland, water area, and bottomland, covering all the ecological types in the research area. Overall, with a high closure degree, connectivity and uniformity index, the network possessed the functional conditions for stable development. The structure of the ecological network complemented the urban space and agricultural space, which is in line with the new concept of coordinated development of ecological and non-ecological space.

### 5.2. Optimization Strategies

According to the results of the current study, the ecological spatial layout of Zhengzhou Metropolitan Area should develop into a structure of one horizontal and two vertical corridors and four clusters, and the outer ecological circle of the central urban area should shift to the south. The branches of the two vertical ecological belts should converge in the central and southern parts to make up for the lack of ecological space in central and southern Zhengzhou. The network system will help the research area to develop a fully enclosed ecological structure with multiple clusters and green belts, thereby forming a multi-layered ecological space surrounding the main urban area of Zhengzhou.

The eastern and southeastern parts of the Zhengzhou Metropolitan Area contain concentrated areas of high-quality arable land. Using these as an ecological source is an important basis for promoting the construction of a green ecological network in plain areas. However, with the development of the Zhengbian Port Area, it has become obvious that the ecological source land in Zhengzhou–Kaifeng–Lankao and between the Airport Industrial Park and Zhongmu is being occupied by urban elements. This creates an obstacle for optimizing the ecological source structure and improving paddy fields, forests and roads.

In order to prevent the spread of urban “pie” type expansion from destroying ecological benefits, creating urban agricultural circles around cities, building modern agricultural production areas and constructing agricultural development spaces are important measures to form ecological green barriers to improve the overall ecosystem value.

## 6. Discussion

In this paper, an ecological network with nine ecological sources, 58 graded ecological corridors and 70 diverse ecological nodes was constructed. With a pattern of concentrated and contiguous ecological resource areas as well as ecological elements including mountains, waters, forests, lakes, grasslands and paddies, it formed a connected system with a complete structure and a regional ecological pattern with multiple functions. The construction and optimization of ecological networks are conducive to the planning of future ecological space and the formulation of a scientifically based ecological pattern.

The analysis of the ecological structure during the large timespan from 1980 to 2035 can ensure that the network pattern maintains sustainable development by following the law of natural evolution. The ecological risk factors for future spatial changes obtained through the comparison with other studies [17,20,21,22,23,47] and the land forecast and structure analysis in 2030 and 2035 can be used for planning and early warning to improve the ecological security pattern and the feasibility of the ecological network. The three levels of ecological corridors and three types of ecological nodes were divided to obtain a new combination of spatial connections, thus forming an ecological network distribution pattern that included special functions. Under the mechanism where sources, corridors and nodes have a mutual influence, the progressive construction of landscape pattern elements such as blocks, substrates, corridors, islands and networks are beneficial to enhance the precision of the ecological network construction.

This paper describes the ecological network layout for the sustainable development of the Zhengzhou Metropolitan Area, based on existing data. However, there is a lack of analysis of the ecological potential brought about by socio-economic attributes in the application of the simulation results. Therefore, in further research, it is necessary to consider the factors influencing the effect of production and living space changes on ecological patterns. The MCR resistance weights were assigned by the AHP method; however, the method does not comprehensively reflect the weighting results because of the experimental inputs and subjectivity. In future research, it is necessary to strengthen the construction of the future trend evaluation system and introduce scientific models for multi-dimensional and multi-angle scientific analysis.

## Figures and Tables

**Table 1:** Parameter settings of the FLUS−Markov coupling model.

Basic Module of Model	Parameter Configuration	Description and Requirements	Specific Content Setting
Back propagation−artificial neural network	Land Use	The reclassified land raster data was reset according to the land class numbers. The part outside the research area was set to “No Data Value”, and the inside part was set to “Valid Data”.	The three groups of data substituted into the research come from 2010, 2015 and 2020, respectively.
	ANN Training	Land use data, including input, hidden, and output layers, etc. were trained and evaluated.	Uniform Sampling was selected as the sampling model. Sampling Rata was set to 1% of the pixels in the research area selected for sampling. Hidden Layer was set to 12 to ensure the high accuracy of the results, thereby reducing errors. [31]
	Save Path	The output files can be set according to two kinds of research requirements of single accuracy or double accuracy.	Accuracy type was set to Double Accuracy.
	Driving Data	Driving factors’ raster data were introduced to simulate the impact of multiple requirements on land development.	A total of 6 types of driving factors were set, all of which are processed into 5906 × 6133 raster data.
Cellular automata space configuration	Probability Data	Probability files of land suitability development were introduced.	The land class probability results were obtained by the artificial neural network module.
	Restricted Data	The restricted area was set to binary data. The value of 0 was not allowed to be converted, and the value of 1 was allowed.	Rivers and ecological reserves in the research area were the main restricted areas.
	Simulation Setting	The simulation parameters were set in detail. Maximum Number of Iterations was 300 times. Neighborhood (odd) was 3 × 3. Accelerate was 0.1. Thread was 8. The Land Use Demand was calculated by the Markov chain. The Cost Matrix was set as a land transfer matrix in the state of natural evolution. Weight of Neighborhood was adjusted according to the simulation results during the simulation [37].	
Markov chain	Predict Year	Divided into the initial year, end year and prediction year	The land data of 2010 and 2015 were used to calculate the land demand in 2030, and the land data of 2015 and 2020 were used to calculate the land demand in 2035.
Accuracy test of results	Accuracy of Kappa	Mathematical analysis was carried out on the accuracy of images of land use spatial layout classification.	The Kappa accuracy of the two periods was 0.702565 and 0.899496, respectively, which was relatively high.
	Accuracy of OA	Overall accuracy was the ratio of the model’s correct prediction number to the total number of all test sets.	The OA accuracy of the two periods was 0.826521 and 0.936027, respectively, which was relatively high.

**Table 2:** MSPA type ecological meaning.

MSPA Elements	Ecological Meaning
Core	The larger green blocks in the foreground land are mostly an important part of the “ecological sources” in the ecological network, and they are often used as habitats or migration sites for species of creatures.
Islet	Small green blocks with weak connectivity or relatively isolated ones are equivalent to “ecological islands” in the ecological network.
Bridge	The natural ecological corridors connecting different core areas have the function of exchanging energy and materials between adjacent core areas.
Branch	The corridors of the MSPA element type connecting the core and non-core areas can exchange materials and energy between the core areas and surrounding landscapes.
Edge	The transition area between the core area and other types of peripheral land can reduce the impact of external factors, protect the ecological function and sustainability of the core area, and take a strong fringe effect.
Loop	The interconnected passages within the same core area are for materials and energy exchange within the core area.
Perforation	Similar to the edge area, the transition area between the core area and the internal non-vegetation type of land has a fringe effect.

**Table 3:** Contribution rate of the connectivity index at different distance thresholds.

Distance Threshold	100 m	500 m	1000 m	1500 m	2000 m
Index	dLCP				
1990	0.61	0.75	0.72	0.72	1.03
2005	0.57	0.96	0.96	0.92	1.26
2020	0.84	0.95	1.42	1.54	1.31
Index	dIIC				
1990	0.55	0.63	0.63	0.63	0.69
2005	0.55	0.78	0.78	0.77	0.85
2020	0.83	0.89	1.09	1.18	1.14
Index	dPC				
1990	0.56	0.70	0.70	0.71	0.76
2005	0.55	0.87	0.92	0.91	0.98
2020	0.83	0.92	1.19	1.33	1.33

**Table 4:** Graded coefficients of ecological resistance.

Resistance Layer	Factor Classification/ Grading	Resistance Value	Resistance Weight	Resistance Layer	Factor Classification/ Grading	Resistance Value	Resistance Weight
MSPA landscape factors	Core	10	0.5638	Land cover types	Paddy field	40	0.2634
	Islet	10			Dry land	50	
	Edge	20			Woodland	10	
	Bridge	20			Grassland	20	
	Branch	30			Water area	30	
	Loop	30			Bottomland	30	
	Perforation	40			Construction land	100	
	Background	80			Unutilized land	60	
Elevation (h)/m	h < 150 m	10	0.1178	Slope (i)/°	i < 5°	10	0.055
	150 m ≤ h < 300 m	20			5° ≤ i < 10°	20	
	300 m ≤ h < 600 m	40			10° ≤ i < 30°	40	
	600 m ≤ h < 1000 m	70			30° ≤ i < 45°	60	
	1000 m ≤ h	90			45° ≤ i	80	

**Table 5:** Land transfer matrix from 1980 to 2035.

1980 to 2035 (km^2^)	Paddy Field	Dry Land	Woodland	Grassland	Water Area	Bottomland	Construction Land	Unutilized Land	Total
Paddy field	—	238.08	4.21	3.18	102.43	2.02	421.29	—	771.21
Dry land	2442.75	—	174.15	214.17	236.06	12.95	4406.21	—	7486.29
Woodland	58.03	405.23	—	73.56	11.92	0.13	202.29	—	751.16
Grassland	—	381.09	141.9	—	14.54	3.55	183.59	—	724.67
Water area	53.02	438.17	4.79	1.1	—	107.09	97.2	1.06	702.43
Bottomland	—	3.22	—	—	0.39	—	1.72	—	5.33
Construction land	433.34	1076.13	16.48	13.64	40.38	0.34	—	—	1580.31
Unutilized land	4.5	4.26	2.49	0.05	0.44	0.23	0.9	—	12.87
Total	2991.64	2546.18	344.02	305.7	406.16	126.31	5313.2	1.06	12,034.27

**Table 6:** Ecological interaction force matrix.

Patch Number	1	2	3	4	5	6	7	8	9	10	11
1	—	5.99	3.75	3.06	4.74	2.24	2.70	2.45	4.15	1.90	1.61
2	—	—	101.81	1.44	2.00	1.10	1.31	1.20	1.88	0.99	0.87
3	—	—	—	1.11	1.50	0.86	1.01	0.93	1.44	0.78	0.70
4	—	—	—	—	1.67	0.95	1.13	1.04	1.60	0.87	0.78
5	—	—	—	—	—	18.59	61.18	35.44	265.43	15.55	9.44
6	—	—	—	—	—	—	11.52	147.38	25.24	109.43	49.75
7	—	—	—	—	—	—	—	22.85	253.24	11.08	6.99
8	—	—	—	—	—	—	—	—	64.39	113.97	33.46
9	—	—	—	—	—	—	—	—	—	23.50	13.17
10	—	—	—	—	—	—	—	—	—	—	116.81
11	—	—	—	—	—	—	—	—	—	—	—

**Table 7:** Ecological corridor hierarchical structure.

Corridor Level	Patch Number	Corridor Length (km)	Corridor Level	Patch Number	Corridor Length (km)
Level—1 corridors	1	455.16	Level—3 corridors	30	43.47
	2	436.14		31	57.28
	3	471.36		32	89.31
	4	18.12		33	96.03
	5	516.24		34	41.49
	6	22.38		35	67.94
	7	88.92		36	65.82
	8	29.22		37	154.74
	9	13.86		38	99.24
	10	333.30		39	172.74
Level—2 corridors	11	455.11		40	38.46
	12	42.60		41	81.90
	13	78.30		42	107.94
	14	81.48		43	96.54
	15	61.98		44	90.84
	16	26.64		45	48.90
	17	38.22		46	44.28
	18	51.06		47	75.96
	19	119.64		48	31.68
	20	17.10		49	61.56
	21	43.92		50	32.46
	22	113.34		51	73.86
	23	38.04		52	151.98
	24	51.24		53	184.50
	25	23.46		54	107.76
	26	84.30		55	178.98
	27	10.66		56	529.02
	28	10.08		57	88.56
	29	55.62		58	246.24

**Table 8:** Evaluation results of ecological network structure index.

	Number of Corridors	Number of Nodes	α Index	β Index	γ Index	Cost Ratio
Level—1 corridors and Strategic nodes	10	10	0.07	1.00	0.42	1.00
Level—2, 3 corridors and natural ecological nodes	48	27	0.45	1.78	0.64	0.99
Level—2, 3 corridors and artificial environmental nodes	48	33	0.26	1.45	0.52	0.99

## Data Availability

The data presented in this study are available on request from the corresponding author.
